# Automated *in vivo* compound screening with zebrafish and the discovery and validation of PD 81,723 as a novel angiogenesis inhibitor

**DOI:** 10.1038/s41598-022-18230-8

**Published:** 2022-08-25

**Authors:** Antonio N. Mauro, Paul J. Turgeon, Sahil Gupta, Koroboshka Brand-Arzamendi, Hao Chen, Jeanie H. Malone, Robin Ng, Kevin Ho, Michelle Dubinsky, Caterina Di Ciano-Oliveira, Christopher Spring, Pamela Plant, Howard Leong-Poi, John C. Marshall, Philip A. Marsden, Kim A. Connelly, Krishna K. Singh

**Affiliations:** 1 Keenan Research Center, Li Ka Shing Knowledge Institute, St. Michael’s Hospital, Unity Health Toronto, Toronto, M5B 1T8 Canada; 2 Institute of Medical Science, University of Toronto, Toronto, M5S 1A8 Canada; 3 Cardiovascular Sciences Collaborative Specialization, University of Toronto, Toronto, M5T 1W7 Canada; 4 Department of Laboratory Medicine and Pathobiology, University of Toronto, Toronto, M5S 1A8 Canada; 5 Faculty of Medicine, School of Medicine, The University of Queensland, Herston, QLD 4006 Australia; 6 Departments of Surgery and Critical Care Medicine, St. Michael’s Hospital, University of Toronto, Toronto, M5B 1W8 Canada; 7 Department of Medical Biophysics, University of Toronto, Toronto, M5G 1L7 Canada; 8 Department of Medicine, University of Toronto, Toronto, M5S 3H2 Canada; 9 Department of Pharmacology and Toxicology, University of Toronto, Toronto, M5S 1A8 Canada; 10 Department of Surgery, University of Toronto, Toronto, M5T 1P5 Canada; 11 Department of Medical Biophysics, Schulich School of Medicine and Dentistry, University of Western Ontario, London, N6A 5C1 Canada

**Keywords:** Drug discovery, Drug screening, High-throughput screening, Angiogenesis

## Abstract

Angiogenesis is a critical process in tumor progression. Inhibition of angiogenesis by blocking VEGF signaling can impair existing tumor vessels and halt tumor progression. However, the benefits are transient, and most patients who initially respond to these therapies develop resistance. Accordingly, there is a need for new anti-angiogenesis therapeutics to delay the processes of resistance or eliminate the resistive effects entirely. This manuscript presents the results of a screen of the National Institutes of Health Clinical Collections Libraries I & II (NIHCCLI&II) for novel angiogenesis inhibitors. The 727 compounds of the NIHCCLI&II library were screened with a high-throughput drug discovery platform (HTP) developed previously with angiogenesis-specific protocols utilizing zebrafish. The screen resulted in 14 hit compounds that were subsequently narrowed down to one, with PD 81,723 chosen as the lead compound. PD 81,723 was validated as an inhibitor of angiogenesis in vivo in zebrafish and in vitro in human umbilical vein endothelial cells (HUVECs). Zebrafish exposed to PD 81,723 exhibited several signs of a diminished endothelial network due to the inhibition of angiogenesis. Immunochemical analysis did not reveal any significant apoptotic or mitotic activity in the zebrafish. Assays with cultured HUVECs elucidated the ability of PD 81,723 to inhibit capillary tube formation, migration, and proliferation of endothelial cells. In addition, PD 81,723 did not induce apoptosis while significantly down regulating p21, AKT, VEGFR-2, p-VEGFR-2, eNOS, and p-eNOS, with no notable change in endogenous VEGF-A in cultured HUVECs.

## Introduction

Angiogenesis is a complex multistep process that requires the tight control and coordination of endothelial cell function. Endothelial cells are retained in a quiescent state in the absence of pro-angiogenesis stimuli. Although, low-level autocrine vascular endothelial growth factor A (VEGF-A) signalling maintains endothelial cell homeostasis^1^. Pathological angiogenesis occurs with a deregulation in angiogenic homeostasis. Insufficient, excessive, and/or abnormal angiogenesis contributes to the pathogenesis of many disorders, such as cancer^2–5^, rheumatoid arthritis^6^, psoriasis^7^, and diabetic retinopathy^8^. In disorders featuring excessive angiogenesis, there is a surplus of pro-angiogenesis factors that may be accompanied by a reduction of angiogenesis inhibitors. Cancer is the most prominent and most studied disease in this category. Tumor progression is dependent on angiogenesis, as uncontrollable cell growth and metastasis cannot be sustained in the absence of neovascularisation^9^.

The idea of targeting pathological angiogenesis to starve tumors was introduced nearly 50 years ago and termed anti-angiogenesis^10^. Several anti-angiogenesis therapies, mainly targeting the VEGF-A/VEGFR-2 signalling axis, have been approved as possible therapeutics for a number of tumor types. However, the benefits are transient, and the vast majority of patients who initially respond to the therapies develop resistance with higher levels of tumor invasiveness and the formation of distant metastasis^11,12^. This indicates a need for new anti-angiogenesis therapeutics that can delay the processes of resistance or eliminate the resistive effects entirely.

The popularity of zebrafish as a well-managed vertebrate model to study human diseases is due to its unique combination of optical clarity as embryos and larvae, and embryological manipulability, which allows for the examination of the onset and progression of a pathological process in vivo and in real time^13^.

The goal of maximizing the high-throughput potential of zebrafish led to the construction and functional development of a high-throughput drug discovery platform (HTP), with the capacity to perform fully automated screens of compound libraries using zebrafish embryos^14^. The pilot project for this prototypical system, in which the National Institutes of Health Clinical Collections Libraries I & II (NIHCCLI&II) were screened for angiogenesis inhibitors, resulted in the discovery of PD 81,723 as a potential angiogenesis inhibitor. This manuscript is a report on the results of this pilot project and the validation of PD 81,723 as an inhibitor of angiogenesis in vivo in zebrafish and in vitro in cultured endothelial cells.

## Results

### Screening the NIH clinical collections libraries I & II

**Figure 1:**
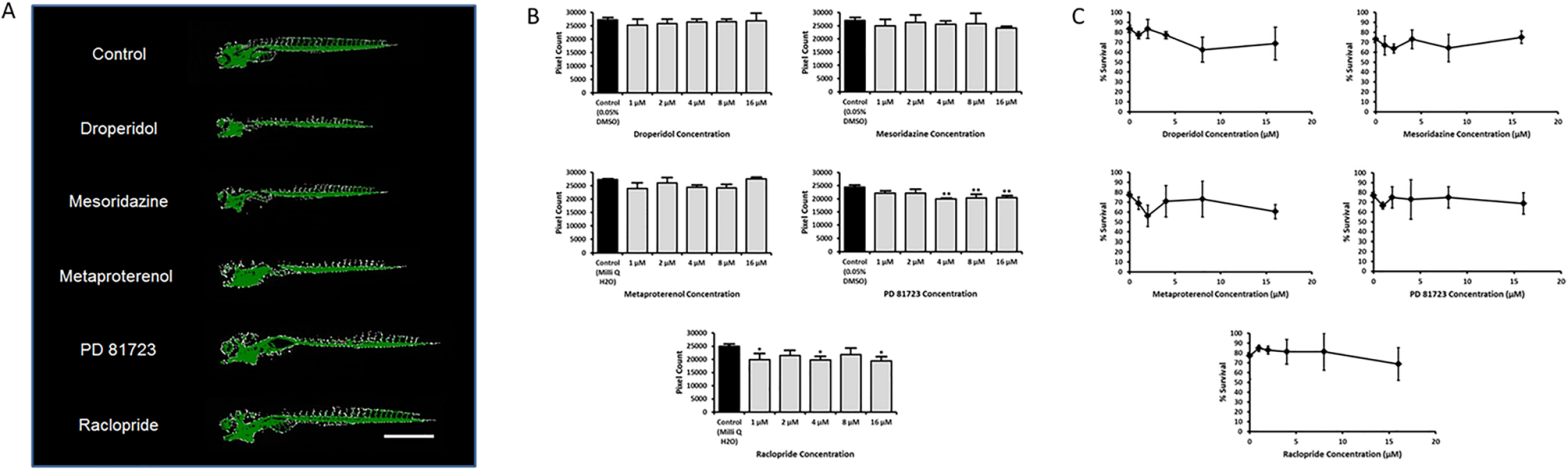
Top 5 hit compounds from a screen of the NIHCCLI&II for angiogenesis inhibitors. (**A**) *Representative images of Tg(kdrl:EGFP) zebrafish treated with the top 5 hit compounds*. Pixels containing endothelial cells are identified and enhanced with the HTP pixel count readout protocol as previously described ^14^. Fish are at 4 dpf. Scale bar is 1 mm. (**B**) *Pixel count readout of Tg(kdrl:EGFP) zebrafish treated with various concentrations of the top 5 compounds compared to a negative control.* PD 81,723 (4, 8, and 16 µM) and raclopride (1, 4, and 16 µM) yielded significantly (** = *P* < 0.01 and * = *P* < 0.05 vs. control) lower pixel counts when compared to the control group. The experiments were repeated in triplicate with an N = 16 for each condition in each experiment. The error bars represent the standard deviation of the means from the three experiments. (**C**) *Survival curves.* None of the compounds seemed to have a major impact on mortality. The error bars represent the standard deviation of the survival percentage from the three experiments.

There were 14 hit compounds from the screen of the NIHCCLI&II libraries for angiogenesis inhibitors. The library was screened 3 times with the HTP protocol described previously^14^ at a concentration of 5 μM for each compound. The compounds were introduced to the embryos at 10–11 h post-fertilization (hpf) and the readouts were taken at 4 days post-fertilization (dpf). The hit compounds are shown in Table 1, ranked according to the standard deviation from the 3 trials. Representative images of the hit compounds, with the pixels containing endothelial cells identified and enhanced, were compared and the 5 compounds that corresponded to the most diminished endothelial network with a relatively normal overall morphology were chosen for further analysis. The representative images of all the hit compounds can be found in Supplementary Fig. S1, with the top 5 hits highlighted. The top 5 compounds were droperidol, mesoridazine, metaproterenol, PD 81,723, and raclopride. Figure 1A shows the representative images of Tg(kdrl:EGFP) zebrafish treated with the top 5 hit compounds compared to a control dosed with 0.05% dimethyl sulfoxide (DMSO).

**Table 1:** The 14 hit compounds from a screen of the NIHCCLI&II libraries for angiogenesis inhibitors.

Rank	Compound	% of negative control mean	Standard deviation (%)
1	Droperidol	65	8
2	3-Trifluoromethylphenylpiperazine (TFMPP)	70	15
3	Mesoridazine	55	21
4	Memantine Hydrochloride	65	23
5	Betaxolol Hydrochloride	65	24
6	Metaproterenol	54	25
7	PD 81,723	53	31
8	Famotidine	61	33
9	Rutin	62	34
10	Lomifylline	70	42
11	CGS 12066B	56	44
12	Raclopride	57	44
13	Mercaptopurine	67	45
14	CGS 15,943	48	51

The library was screened three times. Compounds were considered hits if they reduced the endothelial network by 30% or more on average when compared to the average of the negative control endothelial network. More value was given to consistency; therefore, the compounds were ranked according to the standard deviation between the three replicates.

### Evaluating the top 5 hits

An experiment using the anti-angiogenesis HTP protocol was conducted, with multiple concentrations of each of the top 5 compounds, to determine which compounds would yield a significant result with a higher number of samples. Tg(kdrl:EGFP) zebrafish were distributed 1 fish/well and placed in a 96-well plate, with two columns (N = 16) designated for each concentration. The concentrations of the compounds were 0 µM (vehicle; 0.05% DMSO or Milli-Q water), 1, 2, 4, 8, and 16 µM and the experiment was repeated three times for each compound. Comparing the means for each condition in the three experiments, it was found that PD 81,723 at 4, 8, and 16 µM (*P* < 0.01 vs. control) and raclopride at 1, 4, and 16 µM (*P* < 0.05 vs. control) yielded significantly lower pixel counts when compared to the control group (Fig. 1B). None of the compounds seemed to have a major impact on mortality in these experiments (Fig. 1C).

### Selection of PD 81,723

Due to their ability to significantly reduce the zebrafish endothelial network, PD 81,723 and Raclopride were evaluated further at the concentrations that resulted in a statistically significant result. Fluorescent images were taken at 1, 2, 3, and 4 dpf to monitor the chemical phenotypes due to PD 81,723 (at 4, 8, and 16 µM) and raclopride (at 16 µM). (Supplementary Fig. S2). The fish exposed to PD 81,723 and raclopride exhibited features, such as a hindrance in the development of the caudal vein and artery, diminished EGFP expression in the intersegmental vessels (ISVs) and cranial vasculature, and cardiac edema, that are often observed in the presence of an angiogenesis inhibitor^15–17^. These features were not present in zebrafish dosed with 16 µM raclopride at 3 dpf and 4 dpf. They were only present in the form of a diminished EGFP signal coming from the ISVs in zebrafish dosed with 16 µM PD 81,723. For this reason, in addition to a variable affect that seemed independent to concentration (see Fig. 1B), raclopride was excluded from further study.

**Figure 2:**
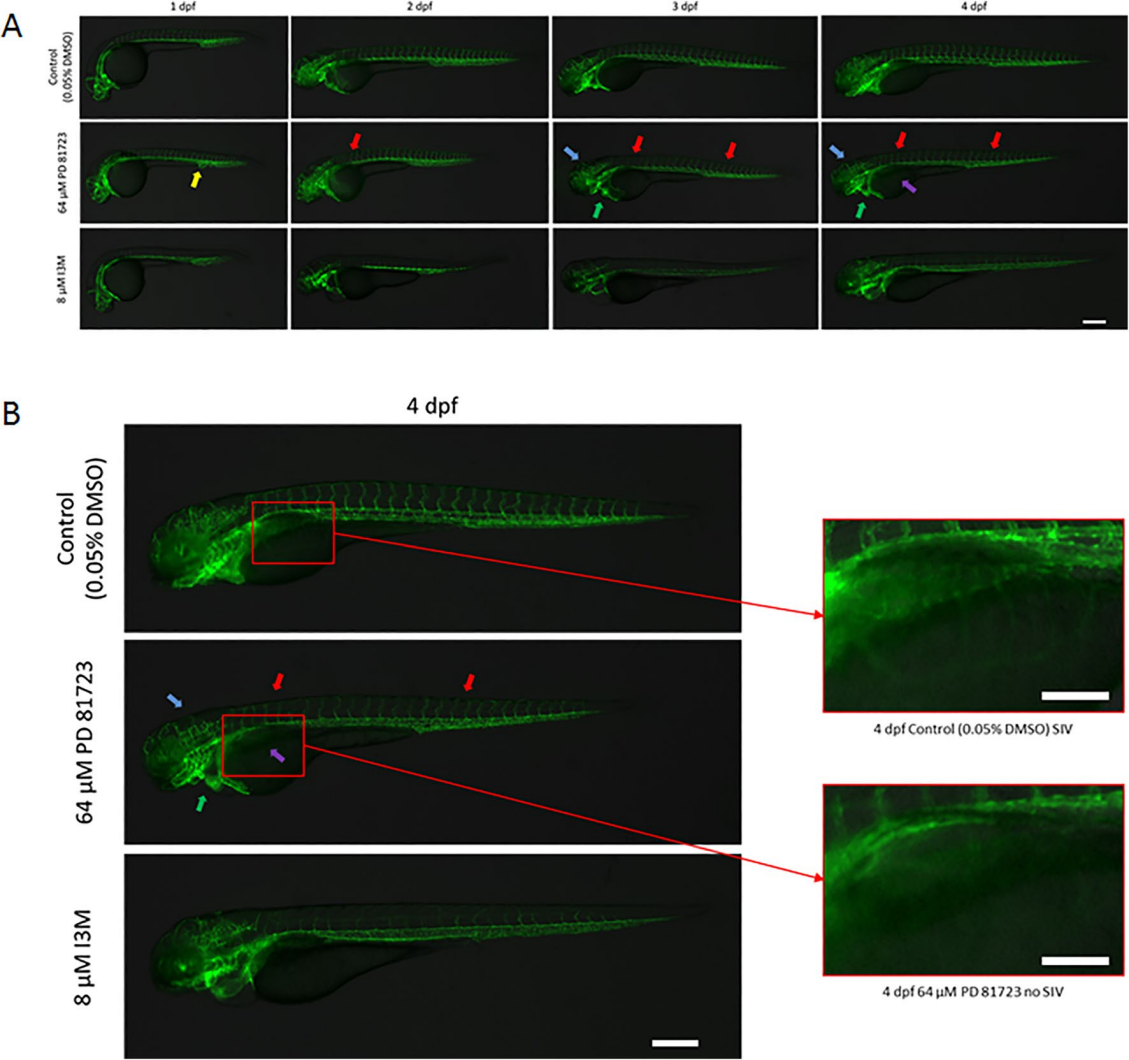
Fluorescent images comparing the vascular phenotype of Tg(kdrl:EGFP) zebrafish treated with 0.05% DMSO, 64 µM PD 81,723, and 8 µM I3M. (**A**) The yellow arrow indicates the area next to the anus where blood islands have formed in the drug dosed fish instead of a mesh-like network that evolves into two separate vessels (caudal artery and caudal vein). The red arrows show a diminished EGFP signal coming from the ISVs. The green arrows are indicating edema surrounding an enlarged heart. The blue arrows are showing a diminished EGFP signal from the cranial vasculature. The purple arrow is pointing to the region where the sub-intestinal vessels (SIVs) have not formed in the fish dosed with a PD 81,723 concentration of 64 µM. Scale bar is 250 µm. (**B**) *The 4 dpf images from A) with the SIV regions of the control and PD 81,723 dosed fish magnified on the right.* The SIVs do not appear to be present in the PD 81,723 dosed fish. Scale bar for the images on the left is 250 µm. Scale bars for the images on the right are 100 µm.

Since, PD 81,723 appeared to be working in a dose-dependent manner; a dose response experiment was conducted with higher concentrations of this compound in order to find the highest concentration that could be used without severely impacting survival. The concentrations of PD 81,723 used were 0 µM (vehicle control; 0.05% DMSO), 16, 32, 64, 128, and 256 µM (N = 16/dose). This experiment was repeated in triplicate using the protocol mentioned previously^14^, with survival as the only readout. This experiment yielded an LD50 concentration of 73.28 µM. With this in mind, 64 µM was chosen as the working concentration of PD 81,723 for further evaluation. Fluorescent images were taken at 1, 2, 3, and 4 dpf to monitor the chemical phenotypes due to PD 81,723 at 64 µM. Figure 2 shows the representative images from this experiment. The fish in this experiment dosed with PD 81,723 at 64 µM exhibited features that are often seen in the presence of an angiogenesis inhibitor that were still evident at the 4 dpf mark. In addition to the diminished EGFP expression in the ISVs and cranial vasculature and cardiac edema, the sub-intestinal vessels (SIVs) were missing in the fish dosed with 64 µM of PD 81,723. Figure 2B highlights the SIV region in the 4 dpf images from Fig. 2A. SIV development has been used in the evaluation of angiogenesis modulators in the past^17^.

### Validation of PD 81,723 as an angiogenesis inhibitor in zebrafish

To confirm the inhibitory effects of PD 81,723 and build a hypothesis for a possible mechanism of action, confocal imaging was employed to construct 3D images of zebrafish at 4 dpf dosed with 64 µM PD 81,723 at 10–11 hpf. Whole body and cranial end images were taken of Tg(kdrl:EGFP) and Tg(Fli1:nEGFP) zebrafish (Supplementary Fig. S3). Images of the whole Tg(kdrl:EGFP) fish body showed results similar to the 2D fluorescent images. They exhibited a diminished EGFP signal coming from the ISVs and cranial vasculature and smaller malformed SIVs when compared to the control. The images on the cranial end of the Tg(kdrl:EGFP) fish, focused on the area around the SIVs, showed a missing vertebral artery (VTA) and parachordal vessel (PAV), a reduction in the vasculature were the ISVs meet the posterior cardinal vein (PCV), and a smaller and malformed SIV network. Images of the whole Tg(fli1:nEGFP) fish body did not show a diminished EGFP signal coming from the ISVs and cranial vasculature as in the Tg(kdrl:EGFP) fish, but smaller malformed SIVs were evident when compared to the control. The images on the cranial end of the Tg(fli1:nEGFP) fish showed nuclei that appeared much larger and less numerous in the PD 81,723 dosed fish when compared to the control.

**Figure 3:**
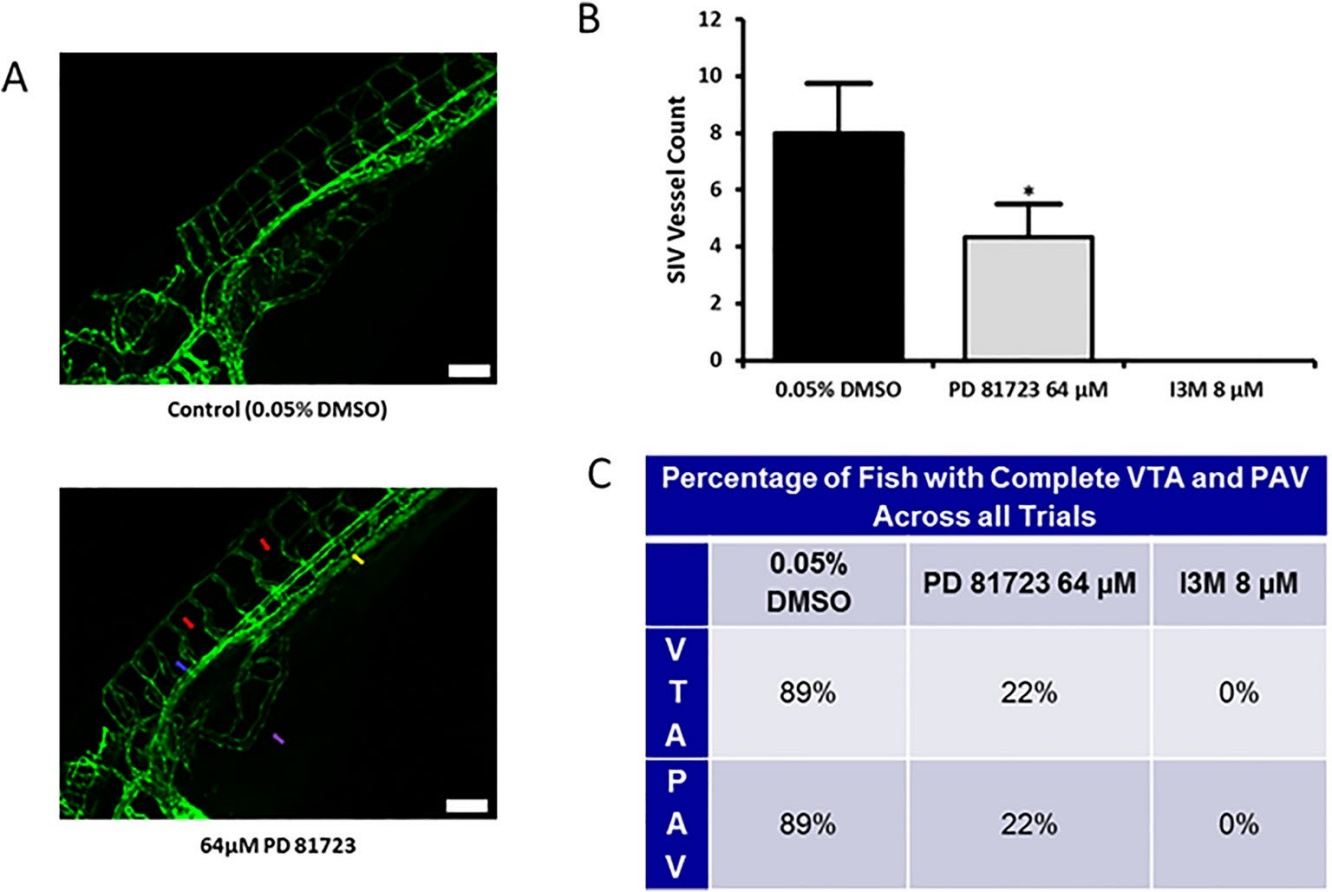
PD 81,723 inhibits the development of SIVs, the VTA, and the PAV. (**A**) Confocal images comparing the vascular phenotype of Tg(kdrl:EGFP) zebrafish at 4 dpf treated with 0.05% DMSO and 64 µM PD 81,723 at the cranial end and focusing on the area surrounding the SIVs. The red arrows indicate a missing vertebral artery (VTA) and the blue arrow indicates a missing parachordal vessel (PAV). The yellow arrow indicates a reduction in the vasculature where the ISVs meet the posterior cardinal vein (PCV). The purple arrow is pointing to the region where the SIVs are malformed. Scale bars are 100 µm. (**B**) SIV counts at 4 dpf in zebrafish dosed with 0.05% DMSO, 64 µM PD 81,723, or 8 µM I3M. There was a significant decrease (* = *P* < 0.05 vs. control) in the number of vessels sprouting from the SIVs basket in the fish dosed with 64 µM of PD 81,723 when compared to the control. The experiment was repeated in triplicate with an N = 3 for each condition in each experiment. The error bars represent the standard deviation of the SIV counts from the three experiments. (**C**) Table showing that only 22% of the fish dosed with PD 81,723 had complete VTA and PAV, compared to 89% in the control, across all three trials.

Based on results of the 3D confocal imaging, the area surrounding the SIVs and the ocular vasculature were selected as the regions of interest for the remaining zebrafish experiments. To quantify the inhibitory effect of PD 81,723 on angiogenesis, 3D confocal imaging focusing on the SIVs area was done with a larger sample size (N = 3 repeated three times), using Tg(kdrl:EGFP) fish. The number of vessels sprouting from the base of the SIVs basket was counted and the presence (or absence) of complete VTA and PAV was observed. There was a significant decrease in the mean number of vessels sprouting from the SIVs basket in the fish dosed with 64 µM of PD 81,723 when compared to the control (*P* < 0.05 vs. control). Only 22% of the fish dosed with PD 81,723 demonstrated complete VTA and PAV, compared to 89% in the control (Fig. 3).

In order to look at the functionality of the SIVs, high framerate time-lapse images of Tg(kdrl:EGFP;GATA-1:DsRed) double transgenic zebrafish were taken with a spinning disc confocal microscope to observe and evaluate blood flow. The erythroid-specific GATA-1 promoter is directing DsRed expression in red blood cells. The resulting flow videos (Supplementary Video S1) showed a significant amount of flow in the base vessel of the SIVs basket and a complete absence of flow in vessels sprouting from the base at the caudal end in both the control and PD 81,723 dosed fish. The flow in vessels sprouting from the base at the rostral end was less significant in the PD 81,723 dosed fish, when compared to the control. However, this may have been due to the underdeveloped nature of the SIVs in the PD 81,723 fish.

**Figure 4:**
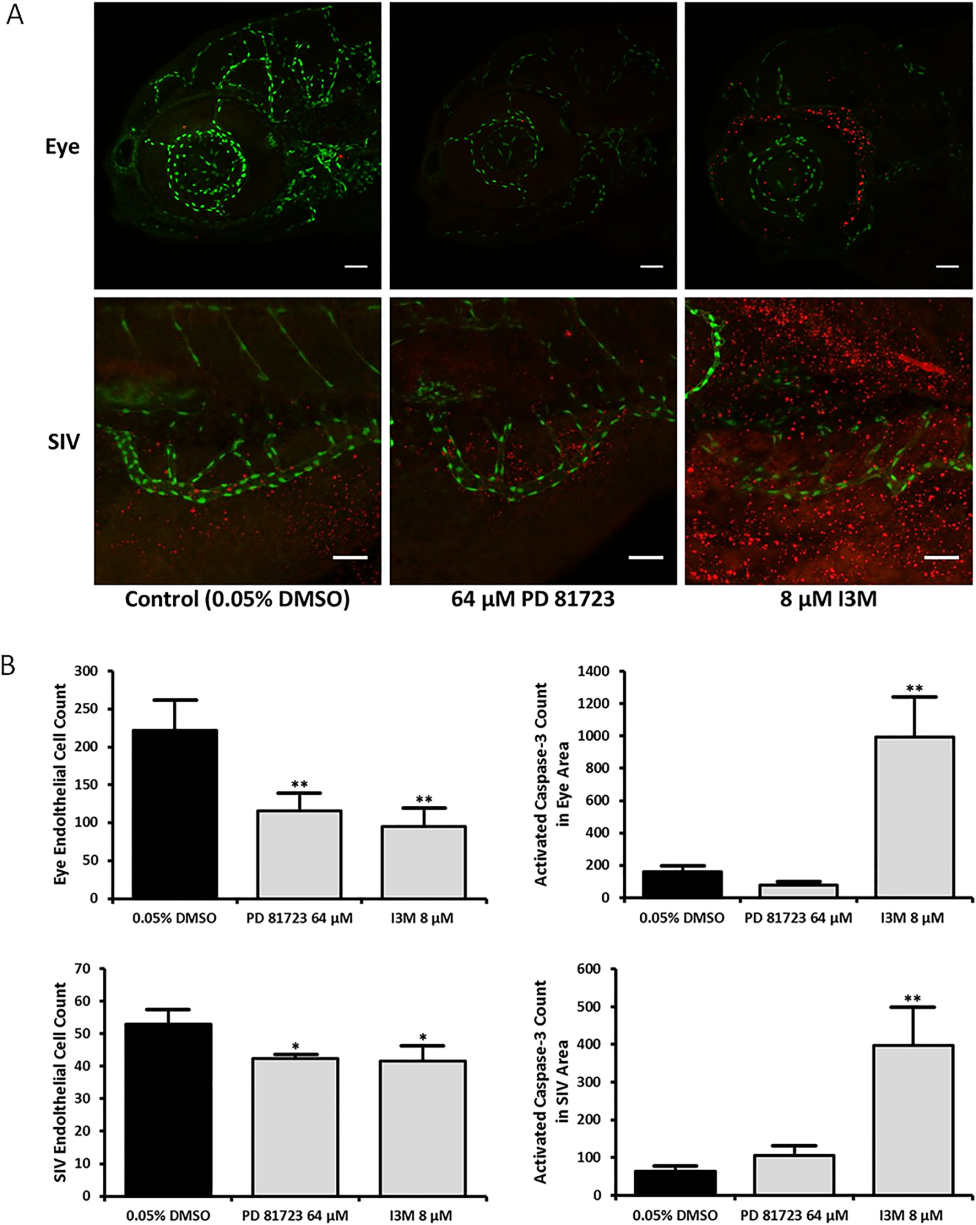
Active Caspase-3 staining in SIVs and ocular area of 4 dpf zebrafish. (**A**) Representative images of SIVs and the ocular area of 4 dpf zebrafish treated with 0.05% DMSO, 64 µM PD 81,723, and 8 μM I3M at 10–11 hpf. Scale bars are 50 µm. (**B**) Graphical representations of the results from three different groups for the caspase-3 stains that were repeated in triplicate with an N = 3 for each group. The error bars represent the standard deviation of the means from the three experiments. The number of endothelial nuclei was significantly reduced in both regions of interest in all instances of this experiment (* = *P* < 0.05 for SIVs area and ** = *P* < 0.01 for eye area vs. control). There was no significant apoptotic activity in the tissue surrounding the ocular vasculature or the SIVs in PD 81,723 fish, when compared to the DMSO group.

**Figure 5:**
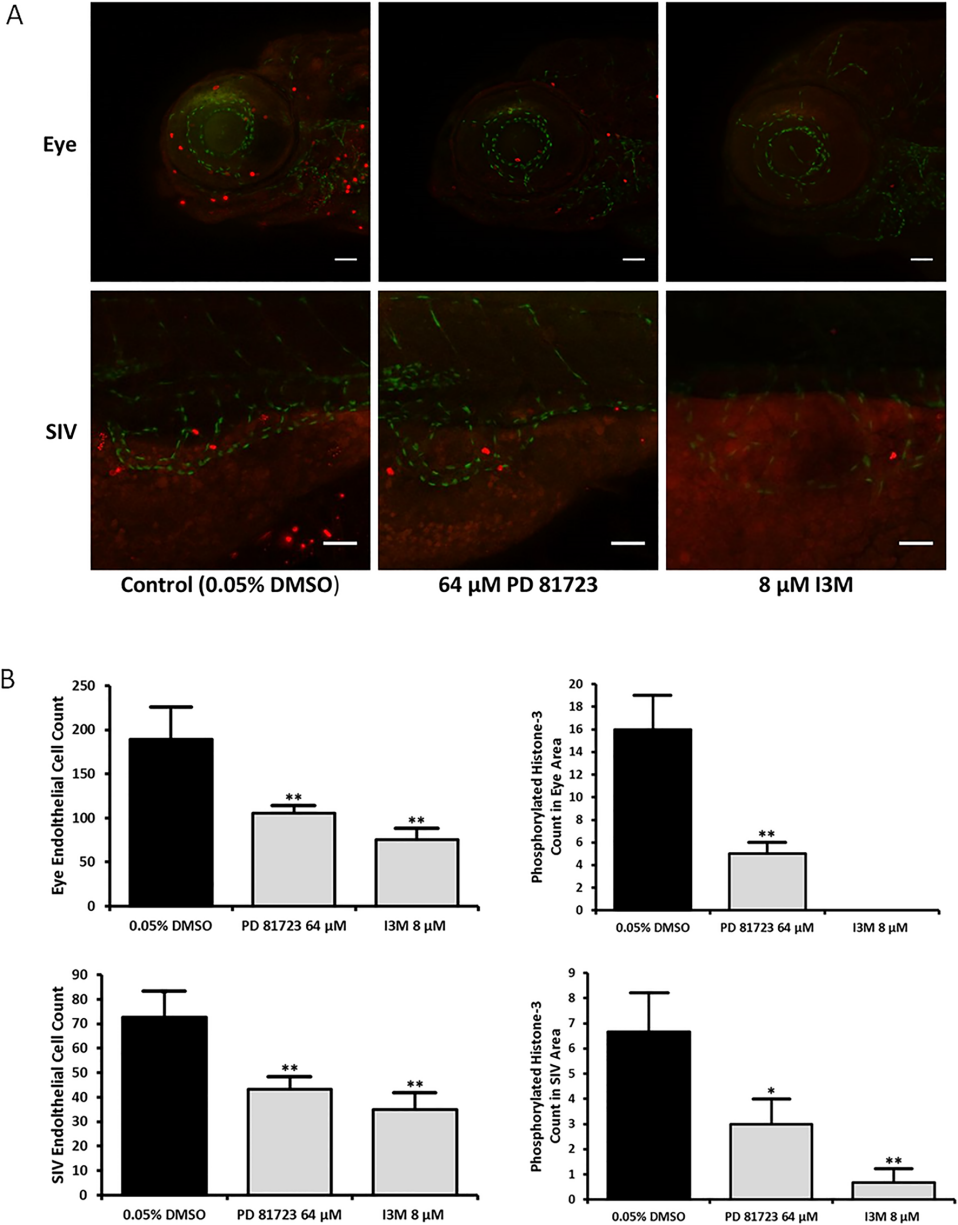
Phosphorylated H3 staining in SIVs and ocular area of 4 dpf zebrafish. (**A**) Representative images of SIVs and the ocular area of 4 dpf zebrafish treated with 0.05% DMSO, 64 µM PD 81,723, and 8 μM I3M at 10–11 hpf. Scale bars are 50 µm. (**B**) Graphical representations of the results from three different groups for the phosphorylated H3 stains that were repeated in triplicate with an N = 3 for each group. The error bars represent the standard deviation of the means from the three experiments. The number of endothelial nuclei was significantly reduced in both regions of interest in all instances of this experiment (** = *P* < 0.01 vs. control). A significant reduction in mitotic activity in the tissue surrounding the ocular vasculature (** = *P* < 0.01 vs. control) and the SIVs (* = *P* < 0.05 vs. control) in PD 81,723 fish was observed, when compared to the DMSO group.

Since PD 81,723 seemed to impact the proliferation of endothelial cells, immunochemistry experiments were performed to look at the effect of PD 81,723 on the apoptotic and mitotic activity in the regions of interest (ocular vasculature and SIVs). Tg(fli1:nEGFP) fish were fixed with paraformaldehyde (PFA) and tagged with antibodies targeted against active caspase-3 and phosphorylated histone H3 (p-Histone H3), an apoptotic and mitotic marker, respectively. The ocular vasculature and SIVs were imaged for each fish (N = 3 repeated three times). The number of endothelial nuclei was significantly reduced in both regions of interest in all instances of this experiment when comparing the means (*P* < 0.05 or *P* < 0.01 vs. control). Significant apoptotic activity was not observed in the tissue surrounding the ocular vasculature or the SIVs in PD 81,723 fish, when compared to the control group (Fig. 4). A significant reduction in the mean mitotic activity in the tissue surrounding the ocular vasculature and the SIVs in PD 81,723 fish was observed, when compared to the control group (*P* < 0.01 and *P* < 0.05 respectively vs. control) (Fig. 5). As anticipated, there was no colocalization observed with the apoptotic marker and the endothelial nuclei because the dying cells would have a very weak (or absent) EGFP signal. Unexpectedly, colocalization was not observed with the mitotic marker and the endothelial nuclei.

### Validation of PD 81,723 as an angiogenesis inhibitor in HUVECs

**Figure 6:**
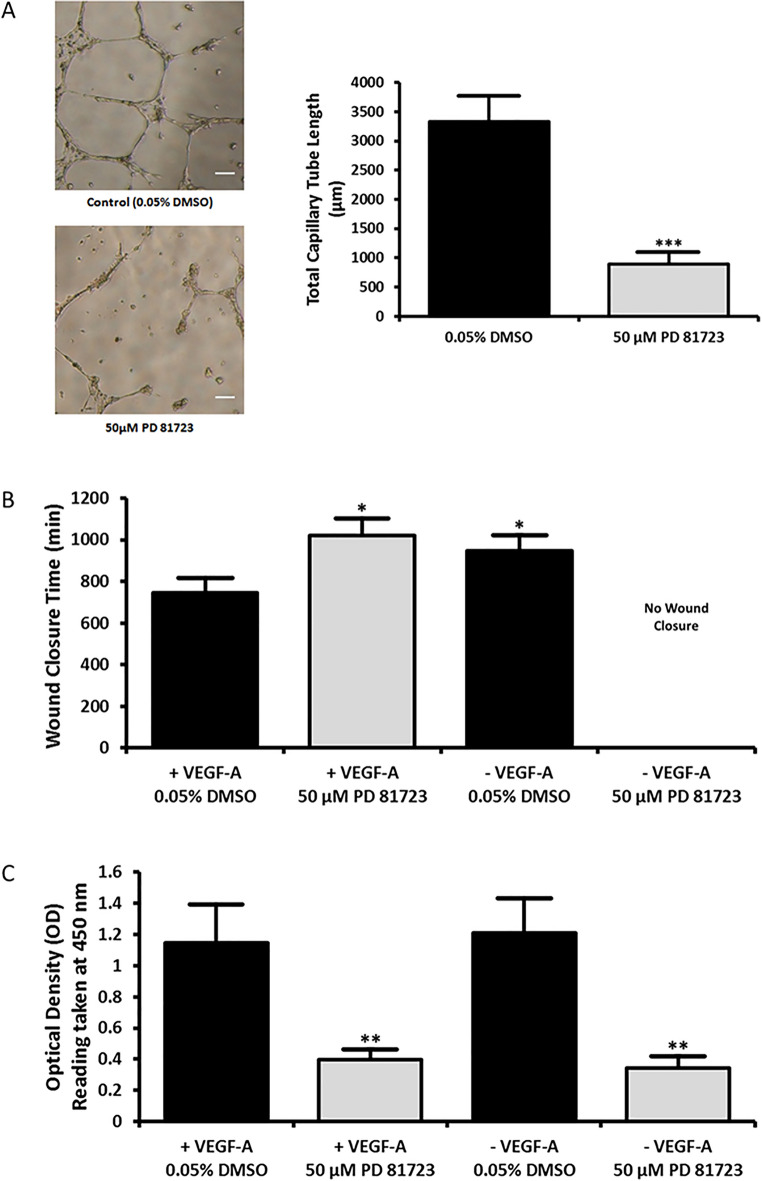
PD 81,723 inhibits angiogenic potential, migration, and cell proliferation. (**A**) *PD 81,723 reduced endothelial branching and outgrowth in a matrigel assay.* Representative images of HUVECs in matrigel treated with 0.05% DMSO and 50 µM PD 81,723 at 9 h after seeding with a graphical representation of the results from the assay. The error bars represent the standard deviation of the means from the six wells for each condition. The total capillary tube length was significantly reduced in the presence of PD 81,723 (*** = *P* < 0.001). Scale bars are 100 µm. (**B**) *PD 81,723 inhibits wound closure in HUVECs.* The cells were treated with 0.05% DMSO or 50 µM PD 81,723 in either complete endothelial growth media or endothelial growth media without VEGF-A. Five sites near the center of each gap were imaged every 30 min for 24 h. The wounds treated with PD 81,723 in complete media and DMSO in media without VEGF-A took significantly longer to close (* = *P* < 0.05) than the wounds treated with DMSO in complete media. The wounds treated with PD 81,723 in VEGF-A deficient media did not close within a 24 h period. The error bars represent the standard deviation of the means from three wells. (**C**) *PD 81,723 inhibits proliferation in HUVECs.* A bromodeoxyuridine (BrdU) incorporation cell proliferation kit was used to look at cell proliferation. The cells were treated with 0.05% DMSO or 50 µM PD 81,723 in either complete endothelial growth media or endothelial growth media without VEGF-A, with five wells per condition in a 96-well plate. There was no notable difference between DMSO groups, while PD 81,723 significantly (** = *P* < 0.01 vs. complete media control) reduced cell proliferation in both cases. This experiment was repeated in triplicate. The error bars represent the standard deviation of the means from the three experiments.

To determine the modulatory effect of PD 81,723 on the endothelial cell phenotype, widely accepted assays of endothelial cell function were conducted, namely matrigel tube formation, wound healing/cell migration, and cell proliferation assays^18,19^. For the matrigel assay, growth factor reduced matrigel was plated onto 96-well plates and seeded with human umbilical vein endothelial cells (HUVECs). Wells were treated with a vehicle control (0.05% DMSO) or 50 µM PD 81,723. Six 1 mm^2^ sites from the center of each well were analyzed for total capillary tube length at 9 h post-seeding. The wells treated with PD 81,723 exhibited a significantly lower average number of capillary tubes when compared to the control (*P* < 0.001) (Fig. 6A).

In the wound healing assay, HUVECs were treated with a control (0.05% DMSO) or 50 µM PD 81,723, in either complete endothelial growth media or endothelial growth media without VEGF-A, with three wells for each condition. The wounds treated with PD 81,723 in complete media and DMSO in media without VEGF-A took significantly longer to close than the wounds treated with DMSO in complete media (*P* < 0.05) (Fig. 6B). The wounds treated with PD 81,723 in VEGF-A deficient media did not close within a 24 h period. Supplementary Video S2 exhibits time-lapse videos representative of each condition over the 24 h.

A bromodeoxyuridine (BrdU) incorporation cell proliferation analysis did not reveal any notable difference in either group treated with a control, while PD 81,723 significantly reduced cell proliferation in both cases when comparing the means from three replicates (*P* < 0.01 vs. vehicle control in complete media) (Fig. 6C).

**Figure 7:**
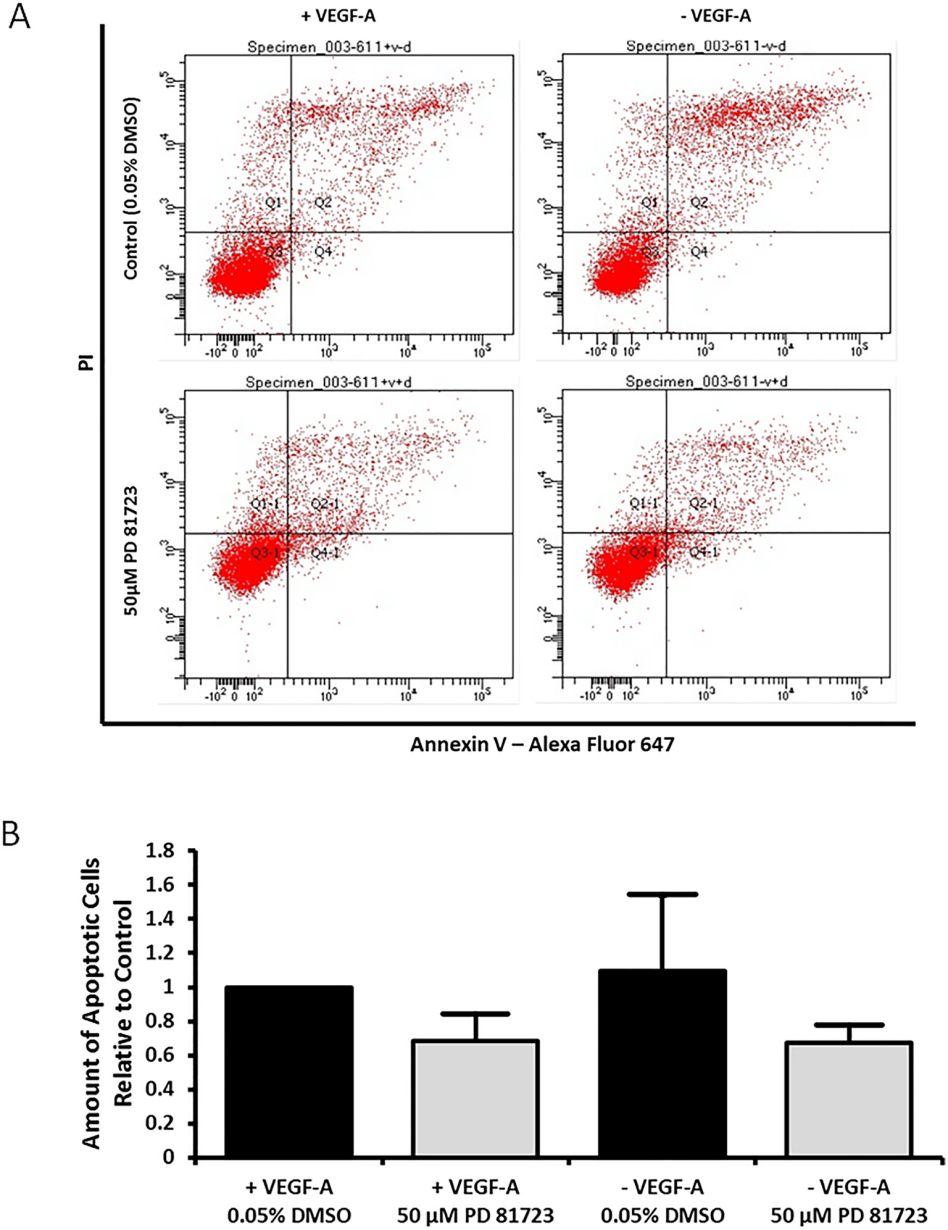
Annexin V/propidium iodide (PI) flow cytometry apoptosis assay with HUVECs. (**A**) Representative scatter plots divided into quadrants showing the distribution of HUVECs marked with annexin V, PI, and double stained with annexin V and PI. The apoptotic activity is quantified by the amount of cells in Q2 and Q2-1. The top plots are controls and the bottom plots are for cells treated with PD 81,723. The plots on the left are for cells grown in complete media and the plots on the right are for cells grown in VEGF-A deficient media. (**B**) Bar graph exhibiting the amount of apoptotic activity for each condition. There was no significant change in the levels of apoptotic events in PD 81,723 treated cells, independent of the addition of VEGF-A to the growth media. This experiment was repeated three times and the error bars represent the standard deviation from three experiments.

An annexin V/propidium iodide (PI) flow cytometry assay was performed to assess apoptotic activity in HUVECs treated with PD 81,723. Figure 7A presents a representative scatter plot divided into quadrants showing the distribution of cells marked with annexin V, PI, and double stained with annexin V and PI. Figure 7B is a bar graph exhibiting the amount of apoptotic activity for each condition. There was no significant change in the levels of apoptotic events in PD 81,723 treated cells, independent of the addition of VEGF-A to the growth media.

**Figure 8:**
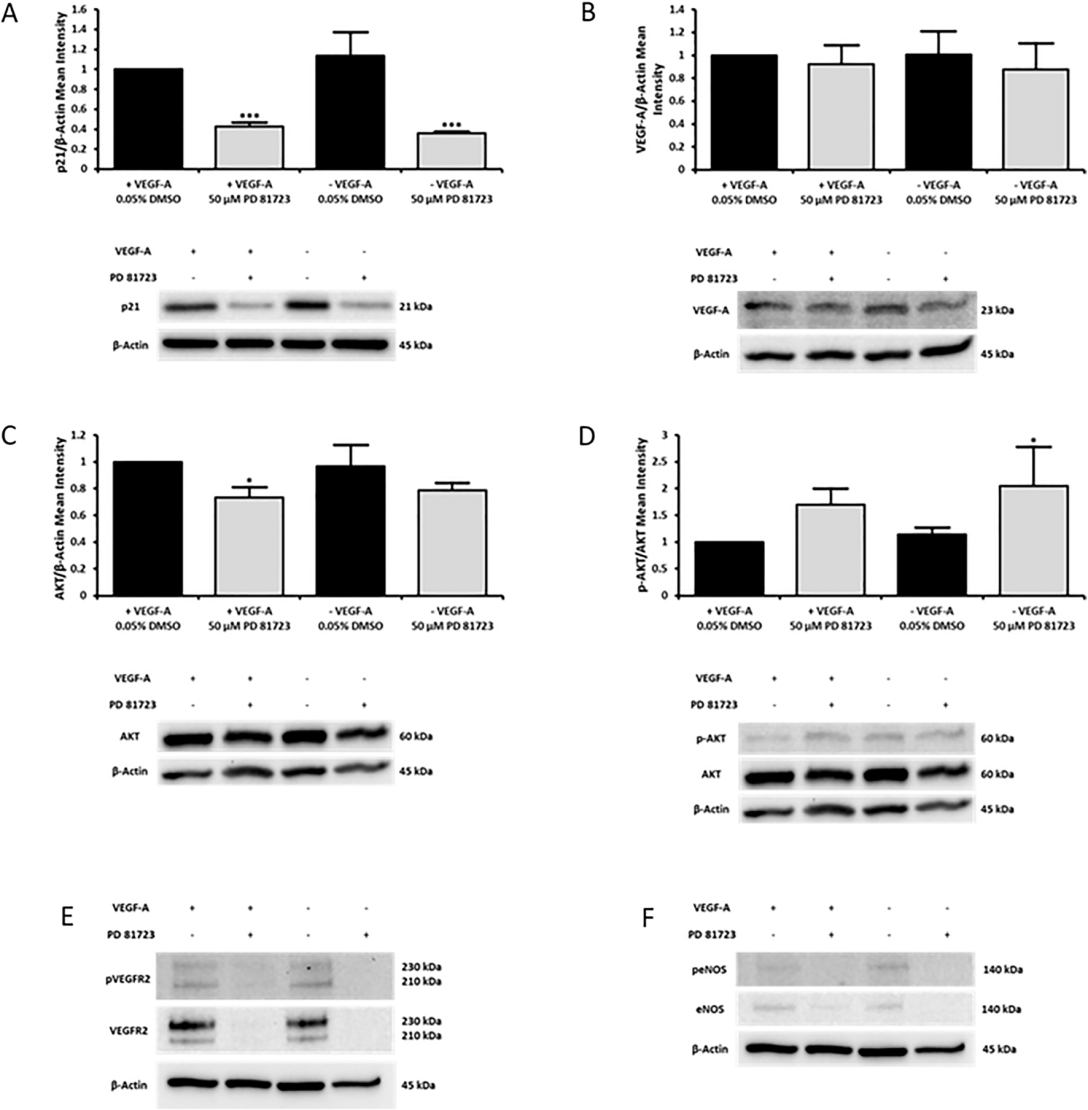
PD 81,723 significantly reduced the expression of p21, AKT, VEGFR-2, and eNOS in HUVECs. HUVECs at 60–70% confluency in 6-well plates were treated with 0.05% DMSO or 50 µM PD 81,723 in either complete endothelial growth media or endothelial growth media deficient in VEGF-A for 24 h. The cells were harvested and protein was extracted 24 h post treatment, with the experiment repeated in triplicate. (**A**) PD 81,723 treated cells expressed significantly (*** = *P* < 0.001 vs. complete media control) lower levels of p21, independent of the addition of VEGF-A in the growth media. (**B**) There was no statistically significant change in endogenous VEGF-A mean protein levels in PD 81,723 treated cells. (**C**) AKT was decreased in the PD 81,723 treated cells; with a statistically significant (* = *P* < 0.05 vs. complete media control) decrease in PD 81,723 treated cells in complete media. (**D**) p-AKT appeared unchanged from observing the blots, but when comparing the ratio of p-AKT/AKT there was an increase in PD 81,723 treated cells; with a statistically significant (* = *P* < 0.05 vs. complete media control) increase in PD 81,723 treated cells in VEGF-A deficient media. (**E**) VEGFR-2 and p-VEGFR-2 did not present any detectable bands in PD 81,723 treated cells. (**F**) The levels of eNOS, and p-eNOS were not enough to present detectable bands in PD 81,723 treated cells. The band intensities for p21, VEGF-A, and AKT were divided by those of the corresponding loading control (β-actin) and p-AKT was divided by the total AKT result. The data was normalized to the results of the 0.05% DMSO treated samples in complete endothelial growth media. The error bars represent the standard deviation from three experiments. The blots for VEGFR2, p-VEGFR2, eNOS, and p-eNOS showed a definitive near absence of protein in PD 81,723 treated cells which did not require mean intensity quantification. Original blots are presented in Supplementary Fig. S4.

In addition, immunoblotting was performed to quantify the levels of key proteins known to play a role in endothelial function, such as p21, VEGF-A, AKT, phosphorylated AKT (p-AKT), VEGFR-2, p-VEGFR-2, eNOS, and p-eNOS. PD 81,723 treated cells expressed significantly lower levels of p21, independent of the addition of VEGF-A in the growth media (*P* < 0.001 vs vehicle control in complete media). Likewise, VEGFR-2, p-VEGFR-2, eNOS, and p-eNOS did not present any detectable bands, indicating reduced expression in PD 81,723 treated cells. There was no statistically significant change in endogenous VEGF-A mean protein levels in PD 81,723 treated cells. AKT was decreased in the PD 81,723 treated cells, with a statistically significant decrease in PD 81,723 treated cells in complete media (*P* < 0.05 vs vehicle control in complete media). While, p-AKT appeared unchanged, the ratio of p-AKT/AKT was increased in PD 81,723 treated cells; with a statistically significant increase in PD 81,723 treated cells in VEGF-A deficient media (*P* < 0.05 vs vehicle control in complete media) (Fig. 8).

## Discussion

The screen of the NIHCCLI&II in this work is a phenotype based screen in which the size of the fluorescent vascular network of Tg(kdrl:EGFP) zebrafish (*Danio rerio*) is quantified in the presence of various compounds. Most small molecule screens in zebrafish are phenotype-based screens, with more than 70 of such screens reported in literature^20,21^. The superiority of the phenotype-based approach is due to several factors: (1) A phenotypic screen can find a compound that can exhibit its efficacy without a validated target. (2) The therapeutic effect of a lead compound from a phenotypic screen can be due to activity at multiple targets. (3) Phenotypic screens can also definitively exclude compounds that cause undesirable outcomes, such as those of highly toxic compounds. Zebrafish screens are typically performed in living embryos or larvae which possess fully integrated vertebrate organ systems, such as functional livers, kidneys, and blood–brain barriers^22–24^. This can provide insight into pharmacological characteristics of the compounds being screened, such as absorption, distribution, metabolism, excretion, and toxicity (ADME-Tox). To invoke or modulate an in vivo phenotype in zebrafish assays, compounds must inherently be absorbed and reach the target tissue, without being rapidly metabolized and/or excreted. This means that compounds discovered in zebrafish screens can be more readily translated to in vivo mammalian models without the extensive optimization of their pharmacological properties^20^. This conserved drug metabolism and ability to evaluate general and organ specific toxicity has been reviewed in the past^25^.

This report presents the discovery of PD 81,723 as potential angiogenesis inhibitor via a fully automated screen of the 727 compounds in the NIHCCLI&II library with subsequent validation in vivo with zebrafish and in vitro with HUVECs. This represents the completion of a successful pilot project for the HTP and serves as further validation for the methods developed for this screen^14^. Zebrafish are ideal for this type of large scale high-throughput screening for chemical and genetic phenotypes because of their small size, optical clarity as embryos and larvae, embryological manipulability, and high fecundity. The strong and definitive fluorescent endothelial cells in the Tg(kdrl:EGFP) fish used in the screens were instrumental in defining and quantifying chemical phenotypes. The lateral symmetry of the endothelial network and strong fluorescent signal of the fish facilitated the quantification of the network and in turn the amount of angiogenesis.

PD 81,723 has been proven as an angiogenesis inhibitor in vivo in zebrafish and in vitro in cultured HUVECs. Tg(kdrl:EGFP) and Tg(Fli1:nEGFP) exposed to PD 81,723 exhibited features that are often seen in the presence of an angiogenesis inhibitor in addition to a reduction in the size and complexity of the overall vascular network, such as a hindrance in the development of the caudal vein and artery, diminished EGFP expression in the ISVs and cranial vasculature, cardiac edema, and a smaller SIVs network. In addition to this, detailed 3D imaging in conjunction with immunochemistry experiments revealed a lower count of endothelial cells and a lack of apoptotic and mitotic activity in the region of those cells when the zebrafish were exposed to PD 81,723. As anticipated, there was no colocalization observed with the apoptotic marker and the endothelial nuclei because the dying cells would have a very weak (or absent) EGFP signal. Unexpectedly, colocalization was not observed with the mitotic marker and the endothelial nuclei. It could be inferred that the reduction in mitotic activity in surrounding tissue would likely affect the rate of angiogenesis because angiogenesis signaling is exogenous in nature and proportional to the demands of surrounding tissue. However, nothing could be inferred about the direct impact of PD 81,723 on endothelial cells. This led to the next step, where cultured HUVECs were used to observe the direct effect of PD 81,723 on endothelial cells. HUVECs exposed to PD 81,723 resulted in complimentary results. The main indices of endothelial function (angiogenic potential, migration, and cell proliferation) were significantly reduced in PD 81,723-treated HUVECs when compared to a vehicle control. In addition, an annexin V/PI flow cytometry assay did not reveal significant levels of apoptotic events in PD 81,723-treated cells, independent of the addition of VEGF-A in the growth media. These results fall in line with the immunochemistry experiments looking at active caspase-3 as the apoptotic marker in zebrafish (Fig. 4).

In addition, immunoblotting revealed that PD 81,723 significantly down regulated p21, AKT, VEGFR-2, p-VEGFR-2, eNOS, and p-eNOS, with no notable change in endogenous VEGF-A. PD 81,723 treated cells expressed significantly lower levels of p21, independent of the addition of VEGF-A in the growth media. This was not expected because p21 is reported to be downregulated in proliferating cells^26^. However, in a recent study endothelial cells were shown to express p21 to a greater degree in migrating tips cells where there is a higher level of VEGFR-2 activity^27^. On the other hand, stalk cells with less VEGFR-2 activity and higher Dll4/Notch activity coincided with cell cycle arrest and lower levels of p21^27,28^. This falls in line with the undetectable quantities of VEGFR-2 and p-VEGFR-2 observed in PD 81,723 treated cells. But, given the context-dependant role of p21 in several complex proliferative and non-proliferative pathways, the precise effect of PD 81,723 on p21 expression and its role needs further investigation^29^. There was no significant change in endogenous VEGF-A mean protein levels in PD 81,723 treated cells, which would lay the blame of a malfunctioning VEGF-A/VEGFR-2 signalling cascade on a lack of expression of VEGFR-2. AKT was decreased and p-AKT/AKT was increased in PD 81,723 treated cells relative to the DMSO control in complete media. A major role of AKT in endothelial cells is as a mediator of cell function by phosphorylating eNOS, leading to the production of nitric oxide (NO), which can promote vasodilation, angiogenesis, and cell proliferation^30,31^. The down regulation of AKT may have been nullified by relative upregulation of its phosphorylation.

The results indicate that PD 81,723 may be driving endothelial cells into a quiescent state. Cellular quiescence is a non-proliferating state that is reversible^32^. Generally endothelial cells are kept in a quiescent state in the relative absence of pro-angiogenesis stimuli, with low levels of autocrine VEGF-A signalling helping maintain endothelial cell homeostasis^1^. With VEGFR-2 being significantly downregulated and no significant change in endogenous VEGF-A, the VEGF signalling required to promote a pro-angiogenesis environment is not achievable, but the low levels required for homeostasis may be achievable in the presence of PD 81,723. In addition, the low levels of p21 and lack of additional cell death may also be indicative of cell cycle arrest. This could be significant for cancer treatments, as dormant/quiescent endothelial cells can help keep tumors dormant and inhibit their growth^33,34^.

PD 81,723 is an allosteric potentiator selectively acting at the adenosine A_1_ receptor via agonist-dependent and -independent mechanisms. It is thought to enhance an agonist affinity for A_1_ receptors and increase the half-life (t_½_) of dissociation from them^35–38^. PD 81 723 was one of the key compounds originally identified as an allosteric regulator of A_1_ receptor binding by the Parke-Davis Pharmaceutical Research Division^36,37^. The original compound series was identified from a 300-ligand adenosine A_1_ binding screen of the Parke-Davis compound bank. The 2-amino-3-benzoylthiophene chemical scaffolds were originally synthesized as intermediates for benzodiazepine-like compounds, but the recognition of their ability to modulate adenosine prompted further analysis. Second generation compounds from this study were found to increase the specific binding of [_3_H]*N*_6_-cyclohexyladenosine, a selective A_1_ receptor agonist, to rat brain membranes. As a result, further compounds were synthesized to identify essential structure–activity relationships and PD 81,723 was identified as an analogue with an improved allosteric profile^35–38^.

There have been many studies that associate allosteric potentiators of adenosine receptors and adenosine itself with pro-angiogenesis and mitogenic effects^39–42^. There have also been studies that associate elevated levels of adenosine and adenosine agonists with endothelial cell apoptosis^43,44^. With several other studies citing the ability of PD 81,723 to aid in the protection against ischemia/reperfusion injury, coinciding with a protection against tissue apoptosis, necrosis, and inflammation in dog cardiac tissue, rat hippocampus, and mouse renal tissue just to name a few^38,45–47^. However, there are no studies evaluating the anti-angiogenic potential of PD 81,723. This study is the first to identify PD 81,723 as an angiogenesis inhibitor with validation in vivo with zebrafish and in vitro with HUVECs. While zebrafish and HUVECs both express A_1_ receptors^48,49^, A_1_ receptors may not be highly expressed in HUVECs^48,50^. It is very likely that the results are mainly due to the allosteric interaction of PD 81,723 at the A_1_ receptor, but the possible off target effects of PD 81,723 should be explored further in future work, since binding at the A_1_ receptor may not be able to account for the entirety of the results, due to its lower expression.

The discovery and validation of PD 81,723 has answered the question of whether or not an angiogenesis inhibitor could be identified using a fully automated screen of the NIHCCLI&II library with the HTP. PD 81,723 has been identified and validated as an angiogenesis inhibitor in vivo and in vitro; however, further studies are warranted to validate these findings in higher order animals. In addition, a possible switching at the molecular level needs to be investigated in the future, as the HUVECs treated with PD 81,723 appeared to have a slightly different appearance than the controls (see Supplementary Video S2).

The two main takeaways from this work are that a fully automated screen of the NIHCCLI&II library was successfully performed with the HTP, serving as further validation for the methods developed previously^14^, and the discovery of a potentially novel angiogenesis inhibitor from said screen. This is the first fully automated high content screen with zebrafish, from embryo dispensing to quantified readout. These results and architecture lay down a foundation that the research community can improve and build on by the creation of even more robust systems and protocols conducive to fully automated experiments that could continue on a trend of minimizing human error and saving time that could be utilized for further innovation.

## Materials and methods

### Screening the NIH clinical collections libraries I & II

The NIHCCLI&II libraries come as a set of 727 compounds, most of which have been in phase I-III clinical trials and not represented in other arrayed collections [Evotec, San Francisco, California, USA]. All compounds are commercially available. These compounds are dispersed over ten 96-well plates, with the first and last columns of each plate left empty for controls. Each compound is supplied as a 10 mM solution in 100% DMSO. The working plates for screening are created from these master plates and contain 50 µM solutions of each compound in 0.5% DMSO. The first row of each working plate contains 0.5% DMSO as a negative control and the last row contains 80 µM of I3M, a known angiogenesis inhibitor^14,51^, in 0.5% DMSO as positive control. This results in final concentrations in the sample plate of 0.05% DMSO for the negative controls, 8 µM of I3M in 0.05% DMSO in the positive controls, and 5 µM in 0.05% DMSO for each compound with 10 µL from the wells in the drug plates being added to the 90 µL of PTU/embryo media already in the wells of the sample plates. A concentration of 5 µM was selected based on the results with I3M from the protocol development^14^. It was a concentration below the concentration that caused a significant result. This was done in hopes of finding a compound that could significantly reduce the endothelial network at a lower concentration and thus be less inclined to be toxic.

The drug screening protocol described previously^14^ is repeated in triplicate for each working plate. The readout was a count of each pixel, from a 2D confocal image, comprising the endothelial network of a single Tg(kdrl:EGFP) zebrafish embryo affected by a compound in the library. The compounds were introduced to the embryos at 10–11 h post-fertilization (hpf) and the readouts were taken at 4 days post-fertilization (dpf). The pixel count data for each run is saved in an excel file. Values under 4000 are assumed to be dead embryos and excluded. Embryos that are visibly dead prior to imaging and distorted values due to poor images are also removed. Wells that were excluded are set to zero and the corresponding compounds are removed from further analysis. The pixel count values for each compound are divided by the negative control mean value and multiplied by 100 to get a percentage. The percentages for each run are then averaged and ranked. Compounds that reduced the mean endothelial network by 30% or more, compared to normal controls, were considered hits and ranked according to consistency (i.e. a lower standard deviation resulted in a higher ranking). This cut-off percentage was chosen in hopes of finding a compound that performs better than the positive control (I3M at 8 μM), which reduced the endothelial network by an average of 25%. Representative images of the hit compounds, with the pixels containing endothelial cells identified and enhanced, were compared and the 5 compounds that corresponded to the most diminished endothelial network with a relatively normal overall morphology were chosen for further analysis.

### Fluorescent stereomicroscope imaging

Fluorescent images were taken with a Leica M205 FA stereomicroscope using an EGFP filter [Leica Microsystems Inc., Concord, Ontario Canada] at 1, 2, 3, and 4 dpf to monitor the chemical phenotypes. Unhatched embryos were removed from their chorion manually. The zebrafish were then anesthetised with a 50/50 E2 media/100 ppm clove oil solution and embedded in 2.5% methylcellulose [Sigma-Aldrich, St. Louis, Missouri, USA], a viscous solution that allows the fish to stay in a desired orientation for imaging.

### Confocal imaging

The typical working distance of a high powered objective on a confocal system is less than 0.5 mm and the penetration depth of confocal imaging is roughly in the realm of 300 µm. For this reason, the fish are placed in µ-dishes with a 180 µm thick microscopy plastic bottom [ibidi Gmbh, Munich, Germany] for confocal imaging. The zebrafish are anesthetised with a 50/50 E2 media/100 ppm clove oil solution in the µ-dishes. Most of the solution is removed once the fish are immobilised, leaving solution only in the recessed region of the dishes. Liquefied 1% low melting point agarose is then placed in the dish and the embryo orientation is adjusted as the agarose solidifies. The confocal microscope used for the 3D imaging is a LSM 700 confocal laser scanning microscope [Carl Zeiss Microscopy GmbH, Jena, Germany]. A 5 × objective is used for imaging in which the whole fish is in the field of view. Images are taken throughout the whole depth of visible fluorescence using an EGFP setting with 5 µm between 2D slices and 4 × frame averaging of 512 × 512 frames. A 10 × objective is used for imaging a region of interest. Images are again taken throughout the whole depth of visible fluorescence using an EGFP setting with 2.5 µm between 2D slices and 4 × frame averaging of 512 × 512 frames.

High framerate time-lapse images of Tg(kdrl:EGFP;GATA-1:DsRed) double transgenic zebrafish were taken with a Quorum Spinning Disc Confocal Microscope (Yokogawa X1 head with Borealis) [Quorum Technologies, Puslinch, Ontario, Canada]. The fish were mounted in the same manner as in the LSM 700 imaging. The endothelial tissue was imaged through the whole depth of the visible fluorescence with an EGFP filter and 20 × objective, capturing 100 evenly space 512 × 512 images. This was immediately followed by single plane imaging at the middle cross section of the SIV vessels with a DsRed filter and 20 × objective, capturing 100 images (512 × 512) over 3 s in order to view blood flow. A 3D image of the endothelium was created and a blood flow video, compiled from the 100 time-lapse images, was overlaid on that 3D image with Fiji (open source ImageJ image processing package)^52^, resulting in a video representative of blood cells flowing through the vasculature.

The 2D representations of the 3D confocal images presented in the main and supplementary figures of this article are maximum intensity projections.

### Active caspase-3 and phosphorylated H3 staining

As in all of the zebrafish work, the embryos were treated at 10–11 hpf and left treated until 4 dpf. At 4 dpf, Tg(fli1:nEGFP) fish were fixed with paraformaldehyde (PFA) and tagged with antibodies targeted against active caspase-3 [Cat. No. 559565, BD Pharmingen, San Jose, California, USA] and p-Histone H3 [Cat. No. sc-8656-R, Santa Cruz Biotechnology, Dallas, Texas, USA]. Embryos were fixed in 4% PFA for 20 min. Embryos were then washed 3 times in phosphate buffered saline (PBS) for 5 min each wash. Next, the embryos were dehydrated with 100% methanol (MeOH) at − 20 °C overnight. Rehydration begins with 5 min in 75% MeOH in PBS tween (PBST) followed by 5 min in 50% MeOH and another 5 min in 25% MeOH. Permeabolization begins with acetone for 10 min at − 20 °C, followed by rinsing with PBST three times for 5 min each rinse. This is followed by 2 h of mild shaking at room temperature in a blocking solution of 5% fetal bovine serum (FBS) in PBST plus 2% bovine serum albumin (BSA). The embryos are then incubated with a primary antibody (either rabbit anti-caspase-3 or p-Histone H3) at a dilution of 1:500 in the blocking solution overnight at 4 °C with mild shaking. The next day, embryos are washed 3 times with PBST for 15 min each wash. Next the embryos are incubated with anti-rabbit Alexa Fluor 568 [Cat. No. A-11011, Thermo Fisher Scientific, Waltham, Massachusetts, USA] at 1:500 dilution as a secondary antibody in blocking solution for 2 h at room temperature. Embryos are then washed 3 times with PBST for 15 min each wash. At this point the fish are fixed with 4% PFA at room temperature for 2 h and then washed in PBS 3 times for 5 min each wash. The fish are then stored in embryo media at 4 °C until they are imaged.

The fish are placed in µ-dishes with a 180 µm thick microscopy plastic bottom [ibidi] and fixed in place with 1% low melting point agarose for 3D confocal imaging. The LSM 700 confocal laser scanning microscope [Carl Zeiss Microscopy GmbH] was used. A 20 × objective is used for imaging the region of interest. Images are again taken throughout the whole depth of visible fluorescence using an EGFP setting for endothelial cell nuclei and Alexa Fluor 568 setting for the protein of interest with 1.25 µm between 2D slices and 4 × frame averaging of 512 × 512 frames. The Imaris image processing software [Bitplane Inc., South Windsor, Connecticut, USA] was used to crop out the regions of interest and count the number of endothelial cells and incidences of the protein of interest. The eye and SIV network were cropped according their major and minor axes and depth. The counts were acquired using the Spots tool in Imaris [Bitplane Inc.].

### Tube formation assay

Growth factor reduced matrigel [BD Biosciences] was plated onto 96-well plates (50 µL/well), incubated at 37 °C for 30 min, and seeded with HUVECs at 10,000 cells/well. Six wells were treated with a control (0.05% DMSO), and six wells were treated with 50 µM PD 81,723. Images from the center of each well were taken with a Nikon Eclipse TS100 inverted microscope [Nikon Instruments Inc., Melville, New York, USA] using a 4 × objective. Six 1 mm^2^ sites from the center of each image were analyzed for total capillary tube length at 9 h after seeding. A 3 × 2 grid was created at the center of each image and the length of each branch was measured and summed for each site using the Fiji open source ImageJ image processing package^52^.

### Wound healing/cell migration assay

The wound-healing assay methods were adapted from methods described previously^53^. HUVECs were seeded into 12-well plates with 350,000 cells/well and incubated at 37 °C until near 70–80% confluency. A p200 pipet tip was used to scratch a gap across the center of the wells. The cells were treated with a control (0.05% DMSO) or 50 µM PD 81,723 in either complete endothelial growth media (basal media and SingleQuotes bullet kit with 20 ng/ml VEGF-A) [Lonza, Wakersville, Maryland, USA] or endothelial growth media without VEGF-A, with three wells for each condition. Five sites near the center of each gap were imaged with a Zeiss Axio Observer widefield microscope [Carl Zeiss Microscopy GmbH] every 30 min for 24 h at 10 × magnification. The microscope was equipped with an EC Plan-Neofluar objective lens and environmental control (37 °C and 5% CO2 with humidity), using a Hamamatsu ORCA-R2 camera. All of the images were compiled into a video for each site using the Fiji open source ImageJ image processing package^52^ and the time of complete wound closure was noted.

### Cell proliferation assay

A bromodeoxyuridine (BrdU) incorporation cell proliferation kit [Catalogue #: 2750, Millipore (Canada) Ltd., Etobicoke, Ontario, Canada] was used to look at cell proliferation. HUVECs were treated with a control (0.05% DMSO) or 50 µM PD 81,723 in either complete endothelial growth media or endothelial growth media deficient in VEGF-A, seeding five wells per condition with 20,000 cells in a 96-well plate and subsequently following the manufacturers protocol. Colorimetric optical density (OD) measurements were obtained using a SpectraMax M5e microplate reader [Molecular Devices, San Jose, California, USA] set at 450 nm.

### Flow cytometry

The annexin V/PI flow cytometry assay methods were adapted from methods described previously^54^. HUVECs at 60–70% confluency in 6-well plates were treated with 0.05% DMSO or 50 µM PD 81,723 in either complete endothelial growth media or endothelial growth media deficient in VEGF-A for 24 h. The cells were then harvested, treated with fluorescently conjugated annexin V [Cat. No. 640943, BioLegend, San Diego, California, USA] and PI [Cat. No. 00-6990-50, Thermo Fisher Scientific] according to the manufacturer’s specifications, and evaluated with an LSRFortessa X-20 cell analyser [Becton Dickinson (BD) Biosciences, San Jose, California, USA]. At least 10,000 events were recorded for each sample. Data was analysed with BD FACSDiva Software [BD Biosciences].

### Western blots

HUVECs at 60–70% confluency in 6-well plates were treated with 0.05% DMSO or 50 µM PD 81,723 in either complete endothelial growth media or endothelial growth media deficient in VEGF-A. The cells were harvested and protein was extracted 24 h post treatment, with the experiment repeated in triplicate. Immunoblots were conducted as described previously^55^. Cells were lysed in RIPA lysis buffer (10 mmol/L Tris, pH 7.4; 150 mmol/L NaCl; 5 mmol/L EDTA; 10 mmol/LNaF; 1 mmol/L phenylmethylsulfonyl fluoride; 1 mmol/L Na3VO4; 50 × protease inhibitor). Lysates were resolved on a 10% SDS-PAGE gel and transferred to a nitrocellulose membrane [GE Healthcare Bio-Sciences AB, SE-751 84 Uppsala, Sweden]. The membrane was immunoblotted with primary antibody and bands were visualized using HRP-conjugated (goat anti-mouse or anti-rabbit) secondary antibody. The following antibodies were used: β-actin Antibody [Product #: 4967, Cell Signaling Technology, Danvers, Massachusetts, USA], Anti-p21 antibody [EPR362] [Product Code: ab109520, abcam, Cambridge, Massachusetts, USA], Anti-VEGFA antibody [EP1176Y] [Product Code: ab52917, abcam], AKT Antibody [Product#: 9272, Cell Signaling Technology], Phospho-AKT (Ser473) Antibody [Product#: 9271, Cell Signaling Technology], VEGF Receptor 2 (55B11) Rabbit mAb [Product#: 2479, Cell Signaling Technology], Phospho-VEGF Receptor 2 (Tyr1175) (D5B11) Rabbit mAb [Product#: 3770, Cell Signaling Technology], eNOS Antibody [Product#: 9572, Cell Signaling Technology], and Phospho-eNOS (Ser1177) Antibody [Product#: 9571, Cell Signaling Technology].

The blots were developed using the ChemiDoc Touch Imaging System [Bio-Rad Laboratories, Hercules, California, USA] and the band intensities were quantified using Image Lab [Bio-Rad]. The mean band intensities for p21, VEGF-A, and AKT were divided by those of the corresponding loading control (β-actin) and p-AKT was divided by the total AKT result. The data was normalized to the corresponding results of 0.05% DMSO treated samples in complete endothelial growth media.

### Compounds

Additional supplies of the top 5 hits were ordered from Axon Medchem [Reston, Virginia, USA] (Droperidol—Catalogue ID: 1554) and Sigma-Aldrich [St. Louis, Missouri, USA] (Mesoridazine—Product #: M4068-5MG, Metaproterenol—Product #: M2398-1G, PD 81,723—Product #: P1123-50MG, and Raclopride—Product #: R121-25MG). Indirubin 3’ Monoxime (I3M) was ordered from MilliporeSigma Canada Co. [Product #: I0404, Oakville, Ontario, Canada].

### Zebrafish

Transgenic Tg(kdrl:EGFP), Tg(fli1:nEGFP), and Tg(GATA-1:DsRed) zebrafish were used in this work. The Tg(kdrl:EGFP) line, also known as Tg(Flk1:EGFP), presents with a vascular endothelial-specific kinase insert domain receptor-like protein (kdrl) promoter, also known as vascular endothelial growth factor receptor 2 (VEGFR-2), directing EGFP expression^56^. EGFP is an enhanced green fluorescence protein with a peak excitation wavelength at 488 nm and a maximum emission wavelength at 509 nm^57,58^. Similarly, the Tg(Fli1:nEGFP) line is a line in which the vascular endothelial-specific friend leukemia integration 1 transcription factor (Fli1) promoter is directing EGFP expression, but in the nucleus^59^. The Tg(GATA-1:DsRed) line is a transgenic line in which the erythroid-specific GATA-binding factor 1 (GATA-1) promoter is directing DsRed expression. GATA-1 is an erythroid transcription factor^60^. DsRed is a red fluorescent protein derived from the coral *Discosoma striata* with a peak excitation wavelength at 558 nm and a maximum emission wavelength at 583 nm^61^. Tg(kdrl:EGFP) and Tg(GATA-1:DsRed) fish were crossed to create Tg(kdrl:EGFP;GATA-1:DsRed) double transgenic zebrafish.

The Tg(kdrl:EGFP)^56^, Tg(fli1:nEGFP)^59^, and Tg(GATA-1:DsRed)^60^ lines were housed in the Li Ka Shing Knowledge Institute (St. Michael’s Hospital, Toronto, Ontario, Canada) research vivarium and maintained and staged as previously described^14,62^. All zebrafish experiments were approved by the St. Michael’s Hospital Animal Care Committee (Toronto, Ontario, Canada) under protocol ACC867 and performed in compliance with the ARRIVE guidelines. The fish were housed under a 14:10 h light:dark cycle at 28 °C. Embryos were produced by pair mating and raised in 1 × E3 embryo medium (5 mMNaCl, 0.17 mMKCl, 0.33 mMCaCl2, 0.33 mM MgSO4).

### HUVECs

Primary HUVECs were isolated from multiple independent donors and maintained as described previously^63,64^. Cells were grown in gelatin coated plates and maintained at 37 °C with 5% CO_2_ in a humidified Steri-Cycle CO_2_ Incubator [Model 370, Thermo Fisher Scientific]. Experiments were conducted at cell passage 3 or 4. All experiments were performed according to the relevant guidelines and regulations.

### Statistical analysis

All statistical analyses were performed using a one-way paired ANOVA with a Tukey’s post-hoc analysis except for the matrigel tube formation assay results, where an independent samples t-test was used. P-values of less than 0.05 were considered statistically significant. Statistical analyses were performed using IBM SPSS Statistics [IBM, Armonk, New York, USA].

## Supplementary Information

Supplementary Information 1.

Supplementary Video 1.

Supplementary Video 2.

## Data Availability

The data generated and/or analysed during this work, if not already included in this manuscript, can be obtained from the corresponding author upon request.

## Code availability

Details of the automated anti-angiogenesis HTP protocol and the associated programs can be found in previous work^14^. In addition, any code that was developed in the process of performing this work can be obtained from the corresponding author upon request.
