# Supplementary material for: Automated in vivo compound screening with zebrafish and the discovery and validation of PD 81,723 as a novel angiogenesis inhibitor
Source: Sci Rep. 2022 Aug 25;12:14537. doi: 10.1038/s41598-022-18230-8 (PMC9411172; doi:10.1038/s41598-022-18230-8)
Supplement: Supplementary file 1 — Supplementary Information 1. [file 41598_2022_18230_MOESM1_ESM.pdf]

Automated *in vivo* Compound Screening with Zebrafish and the Discovery and Validation of PD 81723 as a Novel Angiogenesis Inhibitor

Antonio N. Mauro[1, 2, 3, *], Paul J. Turgeon[1, 4], Sahil Gupta[1, 2, 5], Koroboshka Brand-Arzamendi[1], Hao Chen[1, 2, 3], Jeanie H. Malone[1], Robin Ng[1], Kevin Ho[1], Michelle Dubinsky[1, 2], Caterina Di Ciano-Oliveira[1], Christopher Spring[1, †], Pamela Plant[1], Howard Leong-Poi[1, 2, 3], John C. Marshall[1, 2, 6], Philip A. Marsden[1, 2, 3, 4, 7, 8], Kim A. Connelly[1, 2, 3, *], Krishna K. Singh[1, 2, 9, 10, 11, *]

[1] Keenan Research Center, Li Ka Shing Knowledge Institute, St. Michael's Hospital, Unity Health Toronto, Toronto, M5B 1T8, Canada

[2] Institute of Medical Science, University of Toronto, Toronto, M5S 1A8, Canada

[3] Cardiovascular Sciences Collaborative Specialization, University of Toronto, Toronto, M5T 1W7, Canada

[4] Department of Laboratory Medicine and Pathobiology, University of Toronto, Toronto, M5S 1A8, Canada

[5] Faculty of Medicine, School of Medicine, The University of Queensland, Herston, QLD, 4006, Austrailia

[6] Departments of Surgery and Critical Care Medicine, St. Michael's Hospital, University of Toronto, Toronto, M5B 1W8, Canada.

[7] Department of Medical Biophysics, University of Toronto, Toronto, M5G 1L7, Canada

[8] Department of Medicine, University of Toronto, Toronto, M5S 3H2, Canada

[9] Department of Pharmacology and Toxicology, University of Toronto, Toronto, M5S 1A8, Canada

[10] Department of Surgery, University of Toronto, Toronto, M5T 1P5, Canada

[11] Department of Medical Biophysics, Schulich School of Medicine and Dentistry, University of Western Ontario, London, N6A 5C1, Canada

[†] Deceased.

[*] Corresponding Authors
E-mail:
antonio.mauro@mail.utoronto.ca (ANM); Kim.Connelly@unityhealth.to (KAC); krishna.singh@uwo.ca (KKS)

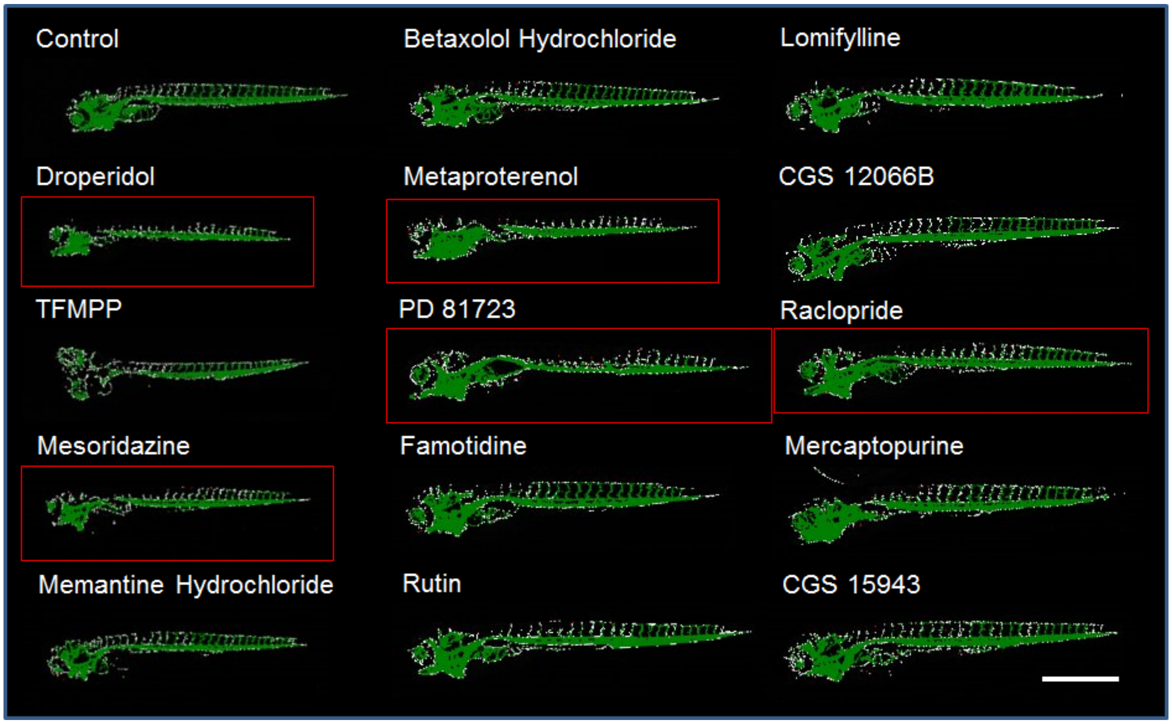

Supplementary Figure S1 – Representative images of all 14 hit compounds with the top 5 hits highlighted in red.
The 5 compounds that corresponded to the most diminished endothelial network with a relatively normal overall morphology were chosen for further analysis. Fish are at 4 dpf. Scale bar is 1mm.

A

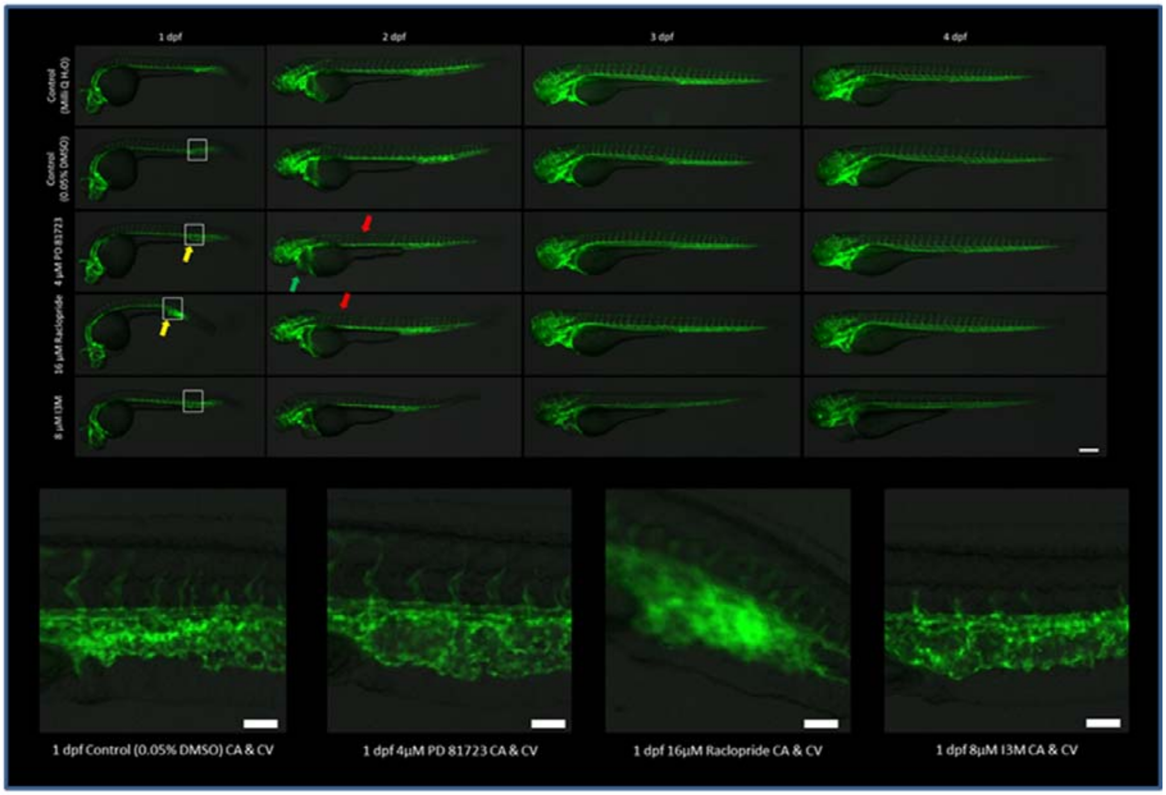

B

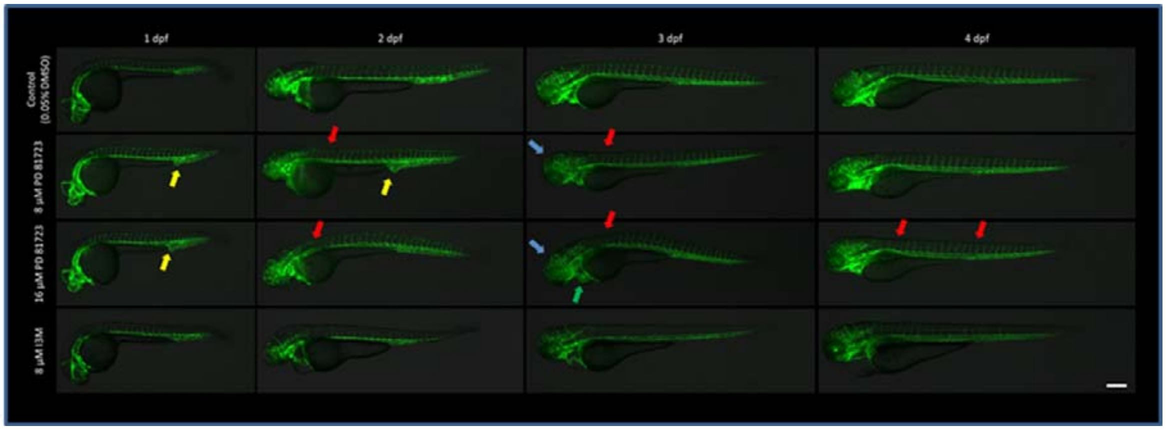

Supplementary Figure S2 – Fluorescent images comparing the vascular phenotype of Tg(kdrl:EGFP) zebrafish treated with Milli-Q water or 0.05% DMSO for vehicle controls, PD 81723, Raclopride, and 8 μM I3M as a positive control.

A) The yellow arrows indicate the area next to the anus where blood islands have formed in the drug dosed fish instead of a mesh-like network that evolves into two separate vessels (caudal artery and caudal vein). The bottom of the figure shows these regions magnified. The red arrows show a diminished EGFP signal coming from the ISVs on the cranial end. The green arrow is indicating edema surrounding an enlarged heart. There are no noticeable differences between the vehicle controls and PD 81723 and raclopride dosed fish at 3 dpf and 4 dpf. Scale bar for the top images is 250 μm. Scale bars for the bottom images are 100 μm. **B)** Again, the yellow arrows indicate the area next to the anus where blood islands have formed in the drug dosed fish. The red arrows show a diminished EGFP signal coming from the ISVs. The green arrow is indicating edema surrounding an enlarged heart. The blue arrows are showing a diminished EGFP signal from the cranial vasculature. The only notable difference by 4 dpf is the slightly diminished signal from the ISVs in the fish dosed with 16 μM PD 81723. Scale bar is 250 μm.

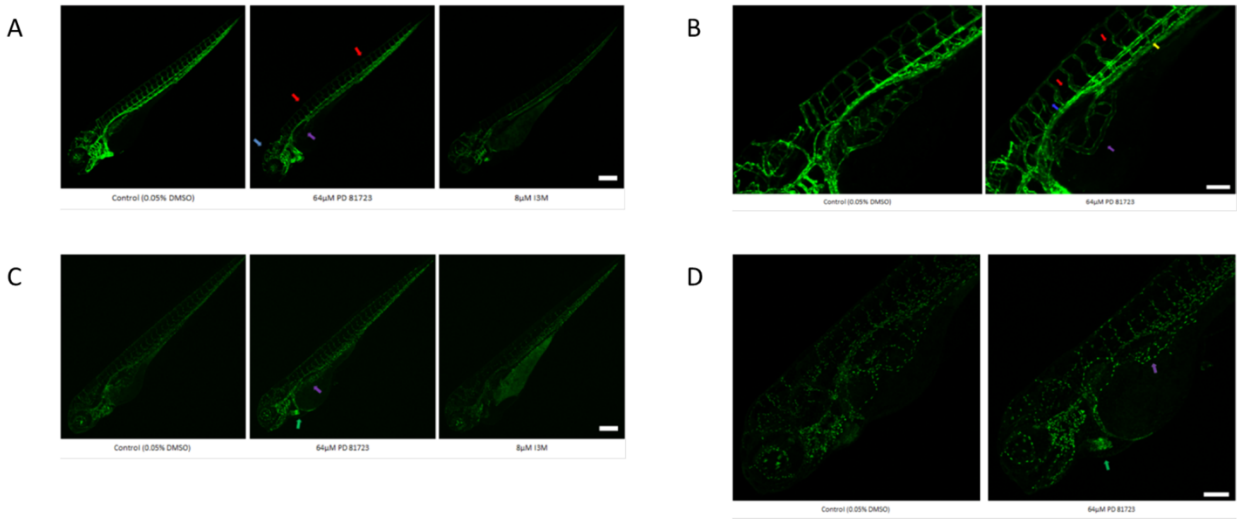

Supplementary Figure S3 – Confocal images comparing the vascular phenotype of Tg(kdrl:EGFP) and Tg(fli1:nEGFP) zebrafish at 4 dpf treated with 0.05% DMSO, 64 µM PD 81723, and 8 µM I3M.
A) *Whole Tg(kdrl:EGFP) fish.* The red arrows indicate a diminished EGFP signal coming from the ISVs. The blue arrow is indicating a diminished EGFP signal from the cranial vasculature. The purple arrow is pointing to the region where the SIVs have not formed well in the fish dosed with a PD 81723 concentration of 64µM. Scale bar is 200 µm. **B)** *Tg(kdrl:EGFP) fish at the cranial end and focusing on the area surrounding the SIVs.* The red arrows indicate a missing vertebral artery (VTA) and the blue arrow indicates a missing parachordal vessel (PAV). The yellow arrow indicates a reduction in the vasculature were the ISVs meet the posterior cardinal vein (PCV). The purple arrow is pointing to the region where the SIVs are malformed. Scale bar is 100 µm. **C)** *Whole Tg(fli1:nEGFP) fish.* The purple arrow is pointing to the region where the SIVs have not formed well in the fish dosed with a PD 81723 concentration of 64µM. The green arrow is indicating edema surrounding an enlarged heart. Scale bar is 200 µm. **D)** *Tg(fli1:nEGFP) fish at the cranial end.* The purple arrow is pointing to the region where the SIVs have not formed well in the fish dosed with a PD 81723 concentration of 64µM. The green arrow is indicating edema surrounding an enlarged heart. The endothelial cell nuclei in the PD 81723 dosed fish seem to be much larger and less numerous than the nuclei in the control. Scale bar is 100 µm.

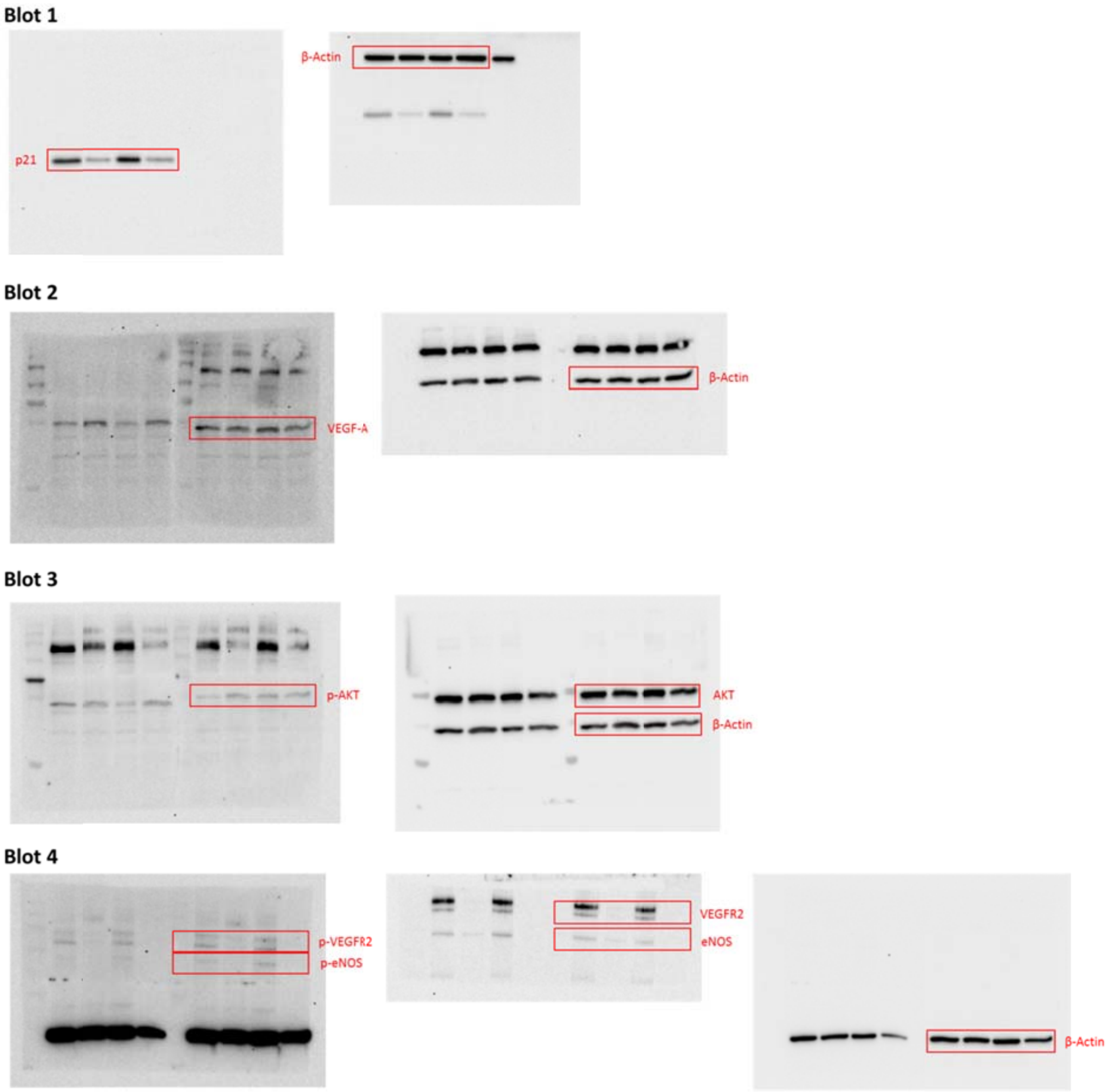

Supplementary Figure S4 – Uncropped representative blot images used for Figure 8
The cropped sections used for Figure 8 are outlined in red and labeled with the corresponding protein. To maximize the use of limited sample quantity the nitrocellulose membranes were cut prior to hybridization with primary antibodies to avoid losing signal to a strip and reprobe approach.

Supplementary Video Captions

Supplementary Video S1 - Time-lapse images of the SIVs in Tg(kdrl:EGFP;GATA-1:DsRed) double transgenic zebrafish.
The video on the left is of a control treated with 0.05% DMSO and the right has a fish treated with 64 µM PD 81723. There is a significant amount of flow in the base vessel of the SIVs basket and a complete absence of flow in vessels sprouting from the base at the caudal end in both the control and PD 81723 dosed fish. The flow in vessels sprouting from the base at the rostral end was not as prominent in the PD 81723 dosed fish, when compared to the control. Scale bars are 50 µm.

Supplementary Video S2 - Time-lapse videos representative of each condition in the wound healing assay with HUVECs.
The wounds treated with PD 81723 in complete media and DMSO in media deficient in VEGF-A took significantly longer to close than the wounds treated with DMSO in complete media (see Figure 6B). The wounds treated with PD 81723 in VEGF-A deficient media did not close within a 24 hr period. Scale bars are 50 µm.
